# Dichlorido{μ_3_-6,6′-dieth­oxy-2,2′-[ethane-1,2-diylbis(nitrilo­methyl­idyne)]diphenolato}octa­methyldi-μ_3_-oxido-tetra­tin(IV)

**DOI:** 10.1107/S1600536809032255

**Published:** 2009-08-19

**Authors:** See Mun Lee, Kong Mun Lo, Seik Weng Ng

**Affiliations:** a Department of Chemistry, University of Malaya, 50603 Kuala Lumpur, Malaysia

## Abstract

In the title tetra­nuclear tin(IV) complex, [Sn_4_(CH_3_)_8_(C_20_H_22_N_2_O_4_)Cl_2_O_2_], there are three completely different tin-atom coordinations. One metal atom (site symmetry 2) adopts a distorted penta­gonal-bipyramidal SnC_2_N_2_O_3_ coordination arising from the *N*,*N*′,*O*,*O*′-tetra­dentate deprotonated Schiff base, two methyl groups in the axial sites and a μ_3_-O atom that also bonds to two further Sn atoms. Two symmetry-equivalent Sn atoms adopt very distorted SnC_2_O_4_ arrangements that could be described as penta­gonal-bipyramidal with one equatorial vertex missing and the C atoms in the axial site. The final Sn atom (site symmetry 2) adopts an SnC_2_Cl_2_O trigonal-bipyramidal arrangement, with Cl atoms in the axial sites. As well as the two Sn atoms, one O atom lies on a twofold rotation rotation axis, and another is disordered about the axis. The terminal eth­oxy group is disordered over two sets of sites with equal occupancy.

## Related literature

For other organotin derivatives of 6,6′-dialk­oxy-2,2′-[ethane-1,2-diylbis(nitrilo­methyl­idyne)]diphenol, see: Cunningham *et al.* (2004 ▶). For the crystal structure of 6,6′-dieth­oxy-2,2′-[ethane-1,2-diylbis(nitrilo­methyl­idyne)]diphenol, see: Ber­mejo *et al.* (2007 ▶).
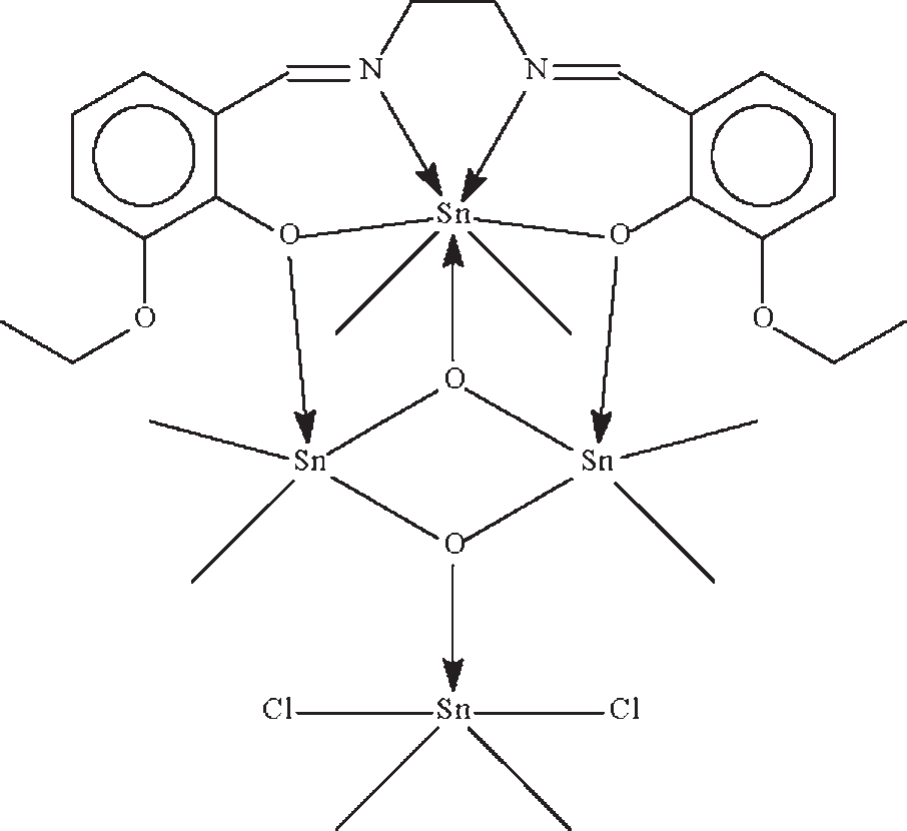


## Experimental

### 

#### Crystal data

[Sn_4_(CH_3_)_8_(C_20_H_22_N_2_O_4_)Cl_2_O_2_]
*M* *_r_* = 1052.33
Tetragonal, 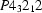

*a* = 9.8723 (1) Å
*c* = 38.0217 (5) Å
*V* = 3705.68 (6) Å^3^
*Z* = 4
Mo *K*α radiation
μ = 2.85 mm^−1^
*T* = 100 K
0.20 × 0.18 × 0.15 mm

#### Data collection

Bruker SMART APEX diffractometer
Absorption correction: multi-scan (*SADABS*; Sheldrick, 1996 ▶) *T*_min_ = 0.863, *T*_max_ = 1.000 (expected range = 0.563–0.652)
68779 measured reflections
4259 independent reflections
4089 reflections with *I* > 2σ(*I*)
*R*_int_ = 0.029

#### Refinement

*R*[*F* ^2^ > 2σ(*F* ^2^)] = 0.027
*wR*(*F* ^2^) = 0.072
*S* = 1.07
4259 reflections
198 parameters
10 restraints
H-atom parameters constrained
Δρ_max_ = 0.42 e Å^−3^
Δρ_min_ = −0.69 e Å^−3^
Absolute structure: Flack (1983 ▶), 1694 Friedel pairs
Flack parameter: 0.00 (4)

### 

Data collection: *APEX2* (Bruker, 2008 ▶); cell refinement: *SAINT* (Bruker, 2008 ▶); data reduction: *SAINT*; program(s) used to solve structure: *SHELXS97* (Sheldrick, 2008 ▶); program(s) used to refine structure: *SHELXL97* (Sheldrick, 2008 ▶); molecular graphics: *X-SEED* (Barbour, 2001 ▶); software used to prepare material for publication: *publCIF* (Westrip, 2009 ▶).

## Supplementary Material

Crystal structure: contains datablocks global, I. DOI: 10.1107/S1600536809032255/hb5035sup1.cif

Structure factors: contains datablocks I. DOI: 10.1107/S1600536809032255/hb5035Isup2.hkl

Additional supplementary materials:  crystallographic information; 3D view; checkCIF report

## Figures and Tables

**Table:** 

Sn1—O3	2.072 (4)
Sn1—C1	2.112 (5)
Sn1—O1	2.410 (3)
Sn1—N1	2.426 (4)
Sn2—O1	2.463 (3)
Sn2—O2	2.791 (4)
Sn2—O3	2.006 (2)
Sn2—C2	2.091 (5)
Sn2—C3	2.100 (5)
Sn2—O4	2.125 (17)
Sn3—O4	1.964 (5)
Sn3—C4	2.114 (5)
Sn3—Cl1	2.5829 (15)

**Table:** 

C1^i^—Sn1—C1	173.9 (3)
C2—Sn2—C3	147.8 (2)
C4—Sn3—C4^i^	132.6 (3)

Symmetry code: (i) 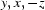.

**Fig. 1.**
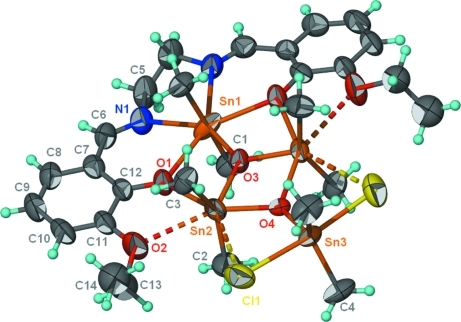
View of (I) at the 70% probability level. Hydrogen atoms are drawn as spheres of arbitrary radius. The disorder in the ethyl groups are not shown. Unlabelled atoms are generated by the symmetry operation (y, x, –z).

## supplementary crystallographic information

### Experimental

One mmol (0.36 g) of the Schiff base was synthesized in toluene according to a literature procedure (Bermejo *et al.*, 2007) from 3-ethoxysalicylaldehyde and ethylenediamine. To the solution was added an excess of triethylamine (0.5 ml). A toluene solution of dimethyltin dichloride (0.20 g, 1 mmol) was added and the mixture heated. Yellow blocks of (I) were isolated from the cool filtered solution.

### Refinement

Hydrogen atoms were placed at calculated positions (C–H 0.95–0.99 Å) and were treated as riding on their parent atoms, with *U*(H) set to 1.2–1.5 times *U*_eq_(C).

The ethoxy group in disordered over two positions in respect of the carbon atoms. The occupancy could not be refined, and was arbitrarily regarded as 0.5 each. The C–O distances were restrained to 1.45±0.01 Å and the C–C distances to 1.50±0.01 Å. The displacement factors of the primed atoms were set of those of the umprimed ones, and the anisotropic temperature factors were restrained to be nearly isotropic by tight restraints.

### Crystal data

**Table:**

[Sn_4_(CH_3_)_8_(C_20_H_22_N_2_O_4_)Cl_2_O_2_]	*D*_x_ = 1.886 Mg m^−^^3^
*M**_r_* = 1052.33	Mo *K*α radiation, λ = 0.71073 Å
Tetragonal, *P*4_3_2_1_2	Cell parameters from 9779 reflections
Hall symbol: P4 nw 2abw	θ = 2.1–26.5°
*a* = 9.8723 (1) Å	µ = 2.85 mm^−^^1^
*c* = 38.0217 (5) Å	*T* = 100 K
*V* = 3705.68 (6) Å^3^	Block, yellow
*Z* = 4	0.20 × 0.18 × 0.15 mm
*F*(000) = 2040	

### Data collection

**Table:**

Bruker SMART APEX diffractometer	4259 independent reflections
Radiation source: fine-focus sealed tube	4089 reflections with *I* > 2σ(*I*)
graphite	*R*_int_ = 0.029
ω scans	θ_max_ = 27.5°, θ_min_ = 2.1°
Absorption correction: multi-scan (*SADABS*; Sheldrick, 1996)	*h* = −12→12
*T*_min_ = 0.863, *T*_max_ = 1.000	*k* = −12→12
68779 measured reflections	*l* = −49→48

### Refinement

**Table:**

Refinement on *F*^2^	Secondary atom site location: difference Fourier map
Least-squares matrix: full	Hydrogen site location: inferred from neighbouring sites
*R*[*F*^2^ > 2σ(*F*^2^)] = 0.027	H-atom parameters constrained
*wR*(*F*^2^) = 0.072	*w* = 1/[σ^2^(*F*_o_^2^) + (0.0361*P*)^2^ + 5.6973*P*] where *P* = (*F*_o_^2^ + 2*F*_c_^2^)/3
*S* = 1.07	(Δ/σ)_max_ = 0.001
4259 reflections	Δρ_max_ = 0.42 e Å^−^^3^
198 parameters	Δρ_min_ = −0.69 e Å^−^^3^
10 restraints	Absolute structure: Flack (1983), 1694 Friedel pairs
Primary atom site location: structure-invariant direct methods	Flack parameter: 0.00 (4)

### Fractional atomic coordinates and isotropic or equivalent isotropic displacement parameters (Å^2^)

**Table:**

	*x*	*y*	*z*	*U*_iso_*/*U*_eq_	Occ. (<1)
Sn1	0.17065 (3)	0.17065 (3)	0.0000	0.05087 (13)	
Sn2	0.33507 (3)	0.46713 (3)	0.035901 (8)	0.04062 (9)	
Sn3	0.64067 (3)	0.64067 (3)	0.0000	0.03517 (10)	
Cl1	0.51581 (18)	0.74108 (17)	0.05370 (4)	0.0693 (4)	
O1	0.1449 (3)	0.3159 (3)	0.05051 (8)	0.0476 (8)	
O2	0.1753 (4)	0.5180 (4)	0.09499 (11)	0.0663 (11)	
O3	0.3190 (3)	0.3190 (3)	0.0000	0.0383 (9)	
O4	0.5075 (16)	0.4933 (15)	0.0032 (5)	0.027 (2)	0.50
N1	−0.0212 (4)	0.0806 (4)	0.03231 (12)	0.0486 (10)	
C1	0.2924 (5)	0.0328 (5)	0.02834 (14)	0.0505 (12)	
H1A	0.3582	−0.0091	0.0124	0.076*	
H1B	0.2350	−0.0376	0.0387	0.076*	
H1C	0.3406	0.0811	0.0471	0.076*	
C2	0.4546 (6)	0.3956 (6)	0.07732 (13)	0.0564 (13)	
H2A	0.5248	0.3352	0.0680	0.085*	
H2B	0.3977	0.3457	0.0940	0.085*	
H2C	0.4974	0.4723	0.0893	0.085*	
C3	0.2074 (5)	0.6200 (5)	0.01652 (15)	0.0509 (12)	
H3A	0.2616	0.7000	0.0105	0.076*	
H3B	0.1406	0.6441	0.0345	0.076*	
H3C	0.1606	0.5873	−0.0045	0.076*	
C4	0.8115 (6)	0.5917 (6)	0.03104 (17)	0.0613 (15)	
H4A	0.8022	0.4991	0.0400	0.092*	
H4B	0.8177	0.6549	0.0508	0.092*	
H4C	0.8936	0.5983	0.0167	0.092*	
C5	−0.0612 (6)	−0.0545 (6)	0.01965 (15)	0.0645 (16)	
H5A	−0.1547	−0.0747	0.0275	0.077*	
H5B	−0.0002	−0.1237	0.0299	0.077*	
C6	−0.0948 (5)	0.1346 (5)	0.05572 (13)	0.0465 (11)	
H6	−0.1747	0.0862	0.0617	0.056*	
C7	−0.0736 (5)	0.2594 (5)	0.07448 (12)	0.0418 (10)	
C8	−0.1778 (5)	0.2942 (6)	0.09830 (14)	0.0509 (12)	
H8	−0.2577	0.2406	0.0992	0.061*	
C9	−0.1654 (6)	0.4048 (6)	0.12023 (14)	0.0575 (14)	
H9	−0.2372	0.4286	0.1357	0.069*	
C10	−0.0475 (6)	0.4810 (6)	0.11964 (12)	0.0551 (14)	
H10	−0.0369	0.5548	0.1354	0.066*	
C11	0.0529 (5)	0.4504 (5)	0.09661 (12)	0.0452 (11)	
C12	0.0447 (5)	0.3385 (5)	0.07272 (11)	0.0399 (9)	
C13	0.2125 (16)	0.6125 (10)	0.1229 (3)	0.061 (2)*	0.50
H13A	0.3109	0.6070	0.1276	0.073*	0.50
H13B	0.1633	0.5900	0.1448	0.073*	0.50
C14	0.1759 (17)	0.7520 (13)	0.1109 (4)	0.080 (3)*	0.50
H14A	0.0782	0.7565	0.1065	0.120*	0.50
H14B	0.2252	0.7734	0.0893	0.120*	0.50
H14C	0.2001	0.8174	0.1293	0.120*	0.50
C13'	0.1933 (14)	0.6412 (9)	0.1157 (3)	0.061 (2)*	0.50
H13C	0.2913	0.6628	0.1162	0.073*	0.50
H13D	0.1656	0.6214	0.1402	0.073*	0.50
C14'	0.1189 (16)	0.7656 (14)	0.1039 (5)	0.080 (3)*	0.50
H14D	0.1808	0.8246	0.0908	0.120*	0.50
H14E	0.0841	0.8141	0.1245	0.120*	0.50
H14F	0.0432	0.7393	0.0887	0.120*	0.50

### Atomic displacement parameters (Å^2^)

**Table:**

	*U*^11^	*U*^22^	*U*^33^	*U*^12^	*U*^13^	*U*^23^
Sn1	0.03415 (14)	0.03415 (14)	0.0843 (3)	−0.00937 (17)	−0.01282 (18)	0.01282 (18)
Sn2	0.04630 (18)	0.03956 (16)	0.03599 (14)	0.01171 (13)	0.00052 (13)	−0.00805 (12)
Sn3	0.03041 (12)	0.03041 (12)	0.0447 (2)	−0.00586 (15)	−0.00722 (12)	0.00722 (12)
Cl1	0.0783 (10)	0.0730 (10)	0.0567 (8)	0.0163 (8)	−0.0115 (7)	−0.0226 (7)
O1	0.0442 (18)	0.0470 (19)	0.0516 (18)	−0.0007 (16)	0.0139 (15)	−0.0160 (15)
O2	0.066 (3)	0.068 (3)	0.065 (2)	−0.014 (2)	0.014 (2)	−0.035 (2)
O3	0.0332 (13)	0.0332 (13)	0.049 (2)	−0.0092 (17)	0.0115 (14)	−0.0115 (14)
O4	0.031 (4)	0.022 (3)	0.029 (5)	−0.004 (3)	−0.006 (3)	0.005 (3)
N1	0.048 (2)	0.039 (2)	0.059 (2)	−0.0105 (17)	0.012 (2)	−0.0063 (19)
C1	0.052 (3)	0.039 (2)	0.061 (3)	0.004 (2)	−0.007 (2)	0.008 (2)
C2	0.058 (3)	0.067 (3)	0.044 (2)	0.004 (3)	−0.007 (2)	0.013 (2)
C3	0.042 (3)	0.041 (3)	0.070 (3)	0.009 (2)	−0.003 (2)	−0.001 (2)
C4	0.044 (3)	0.061 (3)	0.079 (4)	−0.004 (2)	−0.027 (3)	0.011 (3)
C5	0.054 (3)	0.050 (3)	0.090 (4)	−0.023 (2)	0.024 (3)	−0.014 (3)
C6	0.040 (2)	0.049 (3)	0.051 (3)	−0.004 (2)	0.008 (2)	0.006 (2)
C7	0.046 (3)	0.044 (3)	0.035 (2)	0.0085 (19)	0.0034 (19)	0.0063 (19)
C8	0.044 (3)	0.058 (3)	0.051 (3)	0.007 (2)	0.008 (2)	0.008 (2)
C9	0.060 (3)	0.070 (4)	0.043 (3)	0.017 (3)	0.015 (3)	0.003 (2)
C10	0.070 (4)	0.059 (3)	0.036 (2)	0.018 (3)	0.006 (2)	−0.007 (2)
C11	0.051 (3)	0.049 (3)	0.035 (2)	0.009 (2)	0.006 (2)	−0.004 (2)
C12	0.041 (2)	0.048 (3)	0.0302 (19)	0.009 (2)	0.0041 (17)	0.0026 (19)

### Geometric parameters (Å, °)

**Table:**

Sn1—O3	2.072 (4)	C2—H2B	0.9800
Sn1—C1^i^	2.112 (5)	C2—H2C	0.9800
Sn1—C1	2.112 (5)	C3—H3A	0.9800
Sn1—O1	2.410 (3)	C3—H3B	0.9800
Sn1—O1^i^	2.410 (3)	C3—H3C	0.9800
Sn1—N1^i^	2.426 (4)	C4—H4A	0.9800
Sn1—N1	2.426 (4)	C4—H4B	0.9800
Sn2—Cl1	3.310 (2)	C4—H4C	0.9800
Sn2—O1	2.463 (3)	C5—C5^i^	1.497 (12)
Sn2—O2	2.791 (4)	C5—H5A	0.9900
Sn2—O3	2.006 (2)	C5—H5B	0.9900
Sn2—C2	2.091 (5)	C6—C7	1.439 (7)
Sn2—C3	2.100 (5)	C6—H6	0.9500
Sn2—O4	2.125 (17)	C7—C12	1.406 (7)
Sn2—O4^i^	2.192 (16)	C7—C8	1.414 (7)
Sn2—Sn2^i^	3.2943 (6)	C8—C9	1.379 (8)
Sn3—O4	1.964 (5)	C8—H8	0.9500
Sn3—O4^i^	1.964 (5)	C9—C10	1.386 (9)
Sn3—C4	2.114 (5)	C9—H9	0.9500
Sn3—C4^i^	2.114 (5)	C10—C11	1.356 (7)
Sn3—Cl1	2.5829 (15)	C10—H10	0.9500
Sn3—Cl1^i^	2.5829 (15)	C11—C12	1.432 (7)
O1—C12	1.320 (5)	C13—C14	1.493 (9)
O2—C11	1.381 (7)	C13—H13A	0.9900
O2—C13	1.459 (8)	C13—H13B	0.9900
O2—C13'	1.460 (8)	C14—H14A	0.9800
O3—Sn2^i^	2.006 (2)	C14—H14B	0.9800
O4—O4^i^	0.31 (4)	C14—H14C	0.9800
O4—Sn2^i^	2.192 (16)	C13'—C14'	1.499 (9)
N1—C6	1.267 (6)	C13'—H13C	0.9900
N1—C5	1.472 (6)	C13'—H13D	0.9900
C1—H1A	0.9800	C14'—H14D	0.9800
C1—H1B	0.9800	C14'—H14E	0.9800
C1—H1C	0.9800	C14'—H14F	0.9800
C2—H2A	0.9800		
			
O3—Sn1—C1^i^	93.05 (15)	C6—N1—Sn1	130.5 (3)
O3—Sn1—C1	93.04 (15)	C5—N1—Sn1	112.1 (3)
C1^i^—Sn1—C1	173.9 (3)	Sn1—C1—H1A	109.5
O3—Sn1—O1	69.76 (8)	Sn1—C1—H1B	109.5
C1^i^—Sn1—O1	89.99 (17)	H1A—C1—H1B	109.5
C1—Sn1—O1	92.12 (17)	Sn1—C1—H1C	109.5
O3—Sn1—O1^i^	69.76 (8)	H1A—C1—H1C	109.5
C1^i^—Sn1—O1^i^	92.12 (17)	H1B—C1—H1C	109.5
C1—Sn1—O1^i^	89.99 (17)	Sn2—C2—H2A	109.5
O1—Sn1—O1^i^	139.52 (16)	Sn2—C2—H2B	109.5
O3—Sn1—N1^i^	144.20 (10)	H2A—C2—H2B	109.5
C1^i^—Sn1—N1^i^	87.14 (19)	Sn2—C2—H2C	109.5
C1—Sn1—N1^i^	87.92 (18)	H2A—C2—H2C	109.5
O1—Sn1—N1^i^	146.01 (13)	H2B—C2—H2C	109.5
O1^i^—Sn1—N1^i^	74.46 (13)	Sn2—C3—H3A	109.5
O3—Sn1—N1	144.20 (10)	Sn2—C3—H3B	109.5
C1^i^—Sn1—N1	87.92 (18)	H3A—C3—H3B	109.5
C1—Sn1—N1	87.14 (19)	Sn2—C3—H3C	109.5
O1—Sn1—N1	74.46 (13)	H3A—C3—H3C	109.5
O1^i^—Sn1—N1	146.01 (13)	H3B—C3—H3C	109.5
N1^i^—Sn1—N1	71.6 (2)	Sn3—C4—H4A	109.5
O3—Sn2—C2	108.10 (17)	Sn3—C4—H4B	109.5
O3—Sn2—C3	103.75 (16)	H4A—C4—H4B	109.5
C2—Sn2—C3	147.8 (2)	Sn3—C4—H4C	109.5
O3—Sn2—O4	75.7 (4)	H4A—C4—H4C	109.5
C2—Sn2—O4	91.7 (5)	H4B—C4—H4C	109.5
C3—Sn2—O4	100.8 (5)	N1—C5—C5^i^	110.7 (4)
O3—Sn2—O4^i^	74.2 (4)	N1—C5—H5A	109.5
C2—Sn2—O4^i^	99.8 (5)	C5^i^—C5—H5A	109.5
C3—Sn2—O4^i^	93.4 (5)	N1—C5—H5B	109.5
O4—Sn2—O4^i^	8.1 (10)	C5^i^—C5—H5B	109.5
O3—Sn2—O1	69.60 (11)	H5A—C5—H5B	108.1
C2—Sn2—O1	93.19 (18)	N1—C6—C7	128.7 (5)
C3—Sn2—O1	93.30 (17)	N1—C6—H6	115.7
O4—Sn2—O1	144.8 (4)	C7—C6—H6	115.7
O4^i^—Sn2—O1	143.8 (4)	C12—C7—C8	120.0 (5)
O3—Sn2—Sn2^i^	34.82 (9)	C12—C7—C6	125.0 (4)
C2—Sn2—Sn2^i^	105.49 (17)	C8—C7—C6	114.8 (5)
C3—Sn2—Sn2^i^	103.37 (16)	C9—C8—C7	121.0 (5)
O4—Sn2—Sn2^i^	41.0 (4)	C9—C8—H8	119.5
O4^i^—Sn2—Sn2^i^	39.5 (4)	C7—C8—H8	119.5
O1—Sn2—Sn2^i^	104.40 (7)	C8—C9—C10	119.6 (5)
O4—Sn3—O4^i^	9.1 (10)	C8—C9—H9	120.2
O4—Sn3—C4	109.3 (6)	C10—C9—H9	120.2
O4^i^—Sn3—C4	118.2 (6)	C11—C10—C9	120.2 (5)
O4—Sn3—C4^i^	118.2 (6)	C11—C10—H10	119.9
O4^i^—Sn3—C4^i^	109.3 (6)	C9—C10—H10	119.9
C4—Sn3—C4^i^	132.6 (3)	C10—C11—O2	124.1 (5)
O4—Sn3—Cl1	85.2 (6)	C10—C11—C12	122.6 (5)
O4^i^—Sn3—Cl1	87.2 (6)	O2—C11—C12	113.2 (4)
C4—Sn3—Cl1	91.55 (19)	O1—C12—C7	124.0 (4)
C4^i^—Sn3—Cl1	91.49 (19)	O1—C12—C11	119.6 (4)
O4—Sn3—Cl1^i^	87.2 (6)	C7—C12—C11	116.4 (4)
O4^i^—Sn3—Cl1^i^	85.2 (6)	O2—C13—C14	108.0 (10)
C4—Sn3—Cl1^i^	91.49 (19)	O2—C13—H13A	110.1
C4^i^—Sn3—Cl1^i^	91.55 (19)	C14—C13—H13A	110.1
Cl1—Sn3—Cl1^i^	172.42 (8)	O2—C13—H13B	110.1
C12—O1—Sn1	133.6 (3)	C14—C13—H13B	110.1
C12—O1—Sn2	127.8 (3)	H13A—C13—H13B	108.4
Sn1—O1—Sn2	95.76 (11)	O2—C13'—C14'	117.5 (10)
C11—O2—C13	119.8 (7)	O2—C13'—H13C	107.9
C11—O2—C13'	119.0 (7)	C14'—C13'—H13C	107.9
Sn2—O3—Sn2^i^	110.36 (18)	O2—C13'—H13D	107.9
Sn2—O3—Sn1	124.82 (9)	C14'—C13'—H13D	107.9
Sn2^i^—O3—Sn1	124.82 (9)	H13C—C13'—H13D	107.2
O4^i^—O4—Sn3	85.4 (5)	C13'—C14'—H14D	109.5
O4^i^—O4—Sn2	98 (6)	C13'—C14'—H14E	109.5
Sn3—O4—Sn2	131.5 (10)	H14D—C14'—H14E	109.5
O4^i^—O4—Sn2^i^	73 (6)	C13'—C14'—H14F	109.5
Sn3—O4—Sn2^i^	127.4 (9)	H14D—C14'—H14F	109.5
Sn2—O4—Sn2^i^	99.5 (2)	H14E—C14'—H14F	109.5
C6—N1—C5	117.2 (4)		
			
O3—Sn1—O1—C12	162.8 (4)	O3—Sn2—O4—O4^i^	78 (5)
C1^i^—Sn1—O1—C12	69.6 (4)	C2—Sn2—O4—O4^i^	−174 (6)
C1—Sn1—O1—C12	−104.7 (4)	C3—Sn2—O4—O4^i^	−23 (6)
O1^i^—Sn1—O1—C12	162.8 (4)	O1—Sn2—O4—O4^i^	88 (6)
N1^i^—Sn1—O1—C12	−15.3 (6)	Sn2^i^—Sn2—O4—O4^i^	75 (5)
N1—Sn1—O1—C12	−18.3 (4)	O3—Sn2—O4—Sn3	169.5 (13)
O3—Sn1—O1—Sn2	1.46 (6)	C2—Sn2—O4—Sn3	−82.3 (12)
C1^i^—Sn1—O1—Sn2	−91.79 (18)	C3—Sn2—O4—Sn3	67.9 (12)
C1—Sn1—O1—Sn2	93.93 (18)	O4^i^—Sn2—O4—Sn3	91 (6)
O1^i^—Sn1—O1—Sn2	1.46 (6)	O1—Sn2—O4—Sn3	179.8 (5)
N1^i^—Sn1—O1—Sn2	−176.7 (2)	Sn2^i^—Sn2—O4—Sn3	165.9 (17)
N1—Sn1—O1—Sn2	−179.63 (16)	O3—Sn2—O4—Sn2^i^	3.6 (5)
O3—Sn2—O1—C12	−164.5 (4)	C2—Sn2—O4—Sn2^i^	111.9 (6)
C2—Sn2—O1—C12	87.4 (4)	C3—Sn2—O4—Sn2^i^	−98.0 (6)
C3—Sn2—O1—C12	−61.0 (4)	O4^i^—Sn2—O4—Sn2^i^	−75 (5)
O4—Sn2—O1—C12	−175.1 (9)	O1—Sn2—O4—Sn2^i^	13.9 (13)
O4^i^—Sn2—O1—C12	−161.3 (9)	O3—Sn1—N1—C6	19.7 (6)
Sn2^i^—Sn2—O1—C12	−165.7 (3)	C1^i^—Sn1—N1—C6	−72.6 (5)
O3—Sn2—O1—Sn1	−1.51 (7)	C1—Sn1—N1—C6	110.9 (5)
C2—Sn2—O1—Sn1	−109.65 (19)	O1—Sn1—N1—C6	17.9 (5)
C3—Sn2—O1—Sn1	101.95 (18)	O1^i^—Sn1—N1—C6	−163.3 (4)
O4—Sn2—O1—Sn1	−12.1 (9)	N1^i^—Sn1—N1—C6	−160.3 (6)
O4^i^—Sn2—O1—Sn1	1.7 (9)	O3—Sn1—N1—C5	−165.8 (3)
Sn2^i^—Sn2—O1—Sn1	−2.76 (12)	C1^i^—Sn1—N1—C5	101.8 (4)
C2—Sn2—O3—Sn2^i^	−91.27 (17)	C1—Sn1—N1—C5	−74.6 (4)
C3—Sn2—O3—Sn2^i^	93.72 (16)	O1—Sn1—N1—C5	−167.6 (4)
O4—Sn2—O3—Sn2^i^	−4.2 (5)	O1^i^—Sn1—N1—C5	11.1 (5)
O4^i^—Sn2—O3—Sn2^i^	4.1 (5)	N1^i^—Sn1—N1—C5	14.2 (3)
O1—Sn2—O3—Sn2^i^	−177.87 (9)	C6—N1—C5—C5^i^	134.5 (6)
C2—Sn2—O3—Sn1	88.73 (17)	Sn1—N1—C5—C5^i^	−40.7 (7)
C3—Sn2—O3—Sn1	−86.28 (16)	C5—N1—C6—C7	174.7 (5)
O4—Sn2—O3—Sn1	175.8 (5)	Sn1—N1—C6—C7	−11.1 (8)
O4^i^—Sn2—O3—Sn1	−175.9 (5)	N1—C6—C7—C12	−7.7 (8)
O1—Sn2—O3—Sn1	2.13 (9)	N1—C6—C7—C8	177.8 (5)
Sn2^i^—Sn2—O3—Sn1	180.0	C12—C7—C8—C9	−0.4 (7)
C1^i^—Sn1—O3—Sn2	86.70 (15)	C6—C7—C8—C9	174.3 (5)
C1—Sn1—O3—Sn2	−93.30 (15)	C7—C8—C9—C10	−1.8 (8)
O1—Sn1—O3—Sn2	−2.17 (9)	C8—C9—C10—C11	2.7 (8)
O1^i^—Sn1—O3—Sn2	177.83 (9)	C9—C10—C11—O2	−177.8 (5)
N1^i^—Sn1—O3—Sn2	176.03 (19)	C9—C10—C11—C12	−1.5 (8)
N1—Sn1—O3—Sn2	−3.97 (19)	C13—O2—C11—C10	11.1 (9)
C1^i^—Sn1—O3—Sn2^i^	−93.30 (15)	C13'—O2—C11—C10	−8.6 (9)
C1—Sn1—O3—Sn2^i^	86.70 (15)	C13—O2—C11—C12	−165.5 (7)
O1—Sn1—O3—Sn2^i^	177.83 (9)	C13'—O2—C11—C12	174.7 (6)
O1^i^—Sn1—O3—Sn2^i^	−2.17 (9)	Sn1—O1—C12—C7	10.6 (7)
N1^i^—Sn1—O3—Sn2^i^	−3.97 (19)	Sn2—O1—C12—C7	166.8 (3)
N1—Sn1—O3—Sn2^i^	176.03 (19)	Sn1—O1—C12—C11	−168.7 (3)
C4—Sn3—O4—O4^i^	−167 (8)	Sn2—O1—C12—C11	−12.5 (6)
C4^i^—Sn3—O4—O4^i^	13 (9)	C8—C7—C12—O1	−177.7 (4)
Cl1—Sn3—O4—O4^i^	103 (8)	C6—C7—C12—O1	8.1 (7)
Cl1^i^—Sn3—O4—O4^i^	−77 (8)	C8—C7—C12—C11	1.6 (7)
O4^i^—Sn3—O4—Sn2	−97 (9)	C6—C7—C12—C11	−172.6 (5)
C4—Sn3—O4—Sn2	95.4 (12)	C10—C11—C12—O1	178.7 (5)
C4^i^—Sn3—O4—Sn2	−83.7 (12)	O2—C11—C12—O1	−4.6 (7)
Cl1—Sn3—O4—Sn2	5.4 (11)	C10—C11—C12—C7	−0.7 (7)
Cl1^i^—Sn3—O4—Sn2	−174.0 (11)	O2—C11—C12—C7	176.0 (4)
O4^i^—Sn3—O4—Sn2^i^	65 (8)	C11—O2—C13—C14	−96.5 (12)
C4—Sn3—O4—Sn2^i^	−102.3 (10)	C13'—O2—C13—C14	−4(3)
C4^i^—Sn3—O4—Sn2^i^	78.6 (13)	C11—O2—C13'—C14'	−71.7 (14)
Cl1—Sn3—O4—Sn2^i^	167.7 (11)	C13—O2—C13'—C14'	−169 (5)
Cl1^i^—Sn3—O4—Sn2^i^	−11.7 (11)		

Symmetry codes: (i) *y*, *x*, −*z*.
